# Associations of Abdominal Visceral Fat Content and Plasma Adiponectin Level With Intracranial Atherosclerotic Stenosis: A Cross-Sectional Study

**DOI:** 10.3389/fneur.2022.893401

**Published:** 2022-06-22

**Authors:** Fei-Hong Wang, Long-Yan Meng, Tong-Ya Yu, Yan Tan, Hui Quan, Jia-Yu Hu, Qing-Ke Bai, Jun-Chao Xie, Yan-Xin Zhao

**Affiliations:** ^1^Department of Neurology, Shanghai Tenth People's Hospital, Tongji University School of Medicine, Shanghai, China; ^2^Department of Neurology, Pudong New Area People's Hospital, Shanghai, China

**Keywords:** intracranial atherosclerotic stenosis, abdominal obesity, visceral adipose, adiponectin, stroke

## Abstract

**Background:**

Abdominal obesity and adipocytokines are closely related to atherosclerosis, and adiponectin level is considered one of the important clinical indicators. This study aimed to analyze the associations of abdominal visceral fat content and adiponectin level with intracranial atherosclerotic stenosis (ICAS).

**Methods:**

A total of 186 patients were enrolled in this study. Patients were distributed into ICAS and non-ICAS by the degree of artery stenosis. Plasma adiponectin levels and the ratio of visceral adipose tissue (VAT) to subcutaneous adipose tissue (SAT) were measured. The related factors of intracranial atherosclerotic stenosis were determined using multivariable logistic regression analysis.

**Results:**

The VAT/SAT ratio (OR, 26.08; 95% CI, 5.92–114.83; *p* < 0.001) and adiponectin (OR, 0.61; 95% CI, 0.44–0.84; *p* = 0.002) were found to be the independent predictors of ICAS in a multivariable logistic regression analysis. The prevalence of ICAS increased (T1: 27.4%; T2: 50.0%; T3: 75.8%) as the VAT/SAT ratio tertile increased (*p* < 0.001). The prevalence of ICAS decreased (T1: 72.6%; T2: 54.8%; T3: 25.8%) as the adiponectin tertile increased (*p* < 0.001). In ROC curves analysis, VAT/SAT ratio had a sensible accuracy for the prediction of ICAS. The optimal cut-off value of VAT/SAT ratio to predict ICAS in this study was 1.04 (AUC: 0.747; *p* < 0.001; sensitivity: 67.4%; specificity: 74.7%). The optimal adiponectin cutoff was 3.03 ug/ml (AUC: 0.716; *p* < 0.001; sensitivity:75.8%; specificity: 61.5%).

**Conclusion:**

Higher VAT/SAT ratio and lower plasma adiponectin levels were closely related to the increased risk of intracranial atherosclerotic stenosis.

## Introduction

Ischemic stroke caused by intracranial atherosclerotic stenosis is frequent in the Asian, African–American, and Hispanic populations (1). Large artery intracranial occlusive disease (LAICOD) has become the most common subtype of stroke in the world (2). Stroke is the leading cause of death in China, with a high recurrence rate (3). ICAS is an important predictor of stroke and its recurrence (4, 5). Looking for the relevant factors of ICAS is of great significance for the prediction and early intervention of stroke.

Several studies previously investigated traditional risk factors associated with ICAS including sex, age, metabolic syndrome (MetS), hyperlipidemia, hypertension, diabetes, smoking, and hyperhomocysteinemia (6). Lipid metabolism disorders including elevated low-density lipoprotein cholesterol (LDL-C) and cholesterol levels are the most important modifiable factors for increased risk of recurrent stroke and ICAS-related vascular events (4). Obesity is closely related to cardiovascular events (7). However, little is known about the relationship between obesity and intracranial atherosclerosis. Several obesity-related parameters, such as waist circumference, body mass index (BMI), and waist to hip ratio (WHR) have shown a weaker correlation with intracranial atherosclerosis (8). Abdominal obesity may be more likely to cause atherosclerosis than systemic obesity (9). Measurement of central obesity, especially VAT, may be more closely related to intracranial atherosclerosis. The increase of visceral fat and the decrease of subcutaneous fat accumulation may be positively correlated to atherosclerosis (10). Adiponectin, secreted by adipocytes, is a unique adipokine that has anti-inflammatory, antioxidant, anti-atherosclerotic effects and also a significant inhibitory effect on obesity (11–13). A recent study showed that the adiponectin-transfected endothelial progenitor cells protected T2DM rats from cerebral ischemia-reperfusion injury by increasing angiogenesis (14). Previous reports have shown that the levels of circulating adiponectin are reduced in patients with obesity, T2DM, coronary artery diseases, carotid artery stenosis, and atherosclerotic plaques (15, 16). However, there are few investigations on the relationship between adiponectin and intracranial atherosclerosis. Therefore, this study aimed to investigate the relationship between visceral fat, adiponectin, and intracranial atherosclerosis.

## Methods

### Study Design and Participants

This was a single-center cross-sectional study with 226 consecutive patients recruited from September 2018 to June 2019 at Shanghai Tenth People's Hospital. Patients were admitted if they satisfied the following conditions: Age ≥ 45 years, complete abdominal non-enhanced CT (NECT), and CT angiography (CTA)/magnetic resonance angiography (MRA) of cerebral arteries. If the patient was < 45 years old, had acute cerebral infarction, missed key clinical data, had a history of severe abdominal organ disease or previous abdominal surgery, they would be excluded. All patients gave their informed permission. Gender, age, smoking history, low-density lipoprotein cholesterol, diabetes, hypertension, and coronary heart disease were acquired as baseline characteristics of classical cerebrovascular risk factors. The patients fasted overnight and had 5 ml venous blood drawn the next morning. Within 4 h after anticoagulation with 2% EDTA, the blood was centrifuged at 3,000 rpm for 15 min. Plasma was kept at −80°C in a refrigerator. An enzyme-linked immunosorbent assay (ELISA) kit from MultiSciences in China was used to determine the concentration of plasma adiponectin. Self-reported history of hypertension, use of anti-hypertensive medication before admission, and the diagnosis of hypertension after discharge are all considered hypertension. Diabetes mellitus (DM) was identified as a self-reported history of diabetes, diabetes medication, a glycated hemoglobin level of more than 7%, or a diabetes diagnosis at discharge. Smokers were defined as those who smoked for 6 months and had more than one cigarette per day, while heavy drinkers were defined as those whose average daily alcohol consumption was 2 units for men or 1 unit for women (17). For statistical purposes, we referred to some relevant references (18–20) and defined tertiles for adiponectin and VAT/SAT ratio separately as follows: T1, low-adiponectin (0.83–2.09 ug/ml); T2, middle-adiponectin (2.10–3.41 ug/ml); T3, high- adiponectin (3.42–6.38ug/ml); T1, low-VAT/SAT (0.30–0.84); T2, middle-VAT/SAT (0.85–1.13); T3, high-VAT/SAT (1.14–2.06).

### Radiological Imaging

A 16-section NECT (uCT 510, United Imaging Healthcare) was used to perform abdominal CT images (120 kVp, 100 mAs, 5 mm slide thickness). The intracranial arteries were examined using computed tomography angiography (CTA) to see whether there were any atherosclerotic lesions. Participants with renal insufficiency or a contraindication to contrast agents had their cerebral arteries examined with MRA to see if they had atherosclerotic stenosis. MRA was done using a 3.0T Verio Scanner (Siemens, Erlangen, Germany).

The cerebral atherosclerotic stenosis of each major intracranial artery was examined by two professional radiologists who were not given any personal information about the individuals. If a conflict arises, it is handled by consultation. Severe stenosis occurs when the lumen diameter of an intracranial artery is decreased by 50%. A stenosis of 50% or more in the large intracranial arteries, including intracranial segments of the internal carotid artery (ICA), anterior cerebral artery (ACA), middle cerebral artery (MCA), posterior cerebral artery (PCA), basilar artery (BA), and intracranial segments of vertebral artery (VA) was classified as the ICAS. The warfarin–aspirin symptomatic intracranial disease (WASID) study criteria were used to measure intracranial artery stenosis. The percentage of stenosis in an intracranial artery was calculated using the following formula: the percentage of intracranial atherosclerotic stenosis = {1–[(diameter of narrowest segment)/(diameter of normal segment)]} × 100%, where the diameter of the narrowest segment is the diameter of the artery at the site of the most severe stenosis, and diameter of the normal segment is the diameter of the proximal normal artery (21).

### Measurements of the Abdominal Adipose-Relevant Parameters

The abdominal circumference was measured at the level of the navel. At the level of the navel, ImageJ 1.52a (National Institute of Health, USA) was used to calculate VAT, SAT, and total adipose tissue (TAT) (22). The attenuation threshold was −190 to −30 Hu. The region of interest (ROI) for subcutaneous adipose was drawn along the outline of the abdominal skin, and ROI for visceral adipose was drawn along the inner edge of the abdominal muscles and the anterior edge of the spine, and then the full pixel area within the attenuation of the region of interest was measured. TAT was calculated by VAT plus SAT (23). The VAT/SAT ratio was determined using these two parameters. The liver/spleen (L/S) attenuation ratio <1 was used to characterize non–alcoholic fatty liver disease (NAFLD). Regions of interest with an area of over 2 cm^2^ were placed at different axial levels of the liver, while the spleen was in the same axial image correspondingly, vessels and biliary structures in the liver were avoided (22).

### Statistical Analysis

We used the χ^2^ test and Fisher's exact categorical variables test, as well as independent samples t-tests or Mann-Whitney U tests, depending on the distribution of continuous variables, to perform univariate analyses of demographic characteristics, vascular risk factors, laboratory indices, and imaging characteristics of patients in the ICAS and non-ICAS groups. The mean ± standard deviation or median with interquartile range is used to represent continuous variables. The Shapiro–Wilk test was employed to determine the distribution's normality. After examining standard cerebrovascular risk variables in the study, multivariable logistic regression analysis was used to discover the independent risk factors of intracranial atherosclerosis. The connection between abdominal fat-related measures and adiponectin levels was assessed using the Spearman correlation coefficient. Subjects were placed into three groups with the same sample size based on the tertiles of plasma adiponectin level or VAT/SAT. The prevalence of ICAS was evaluated in each tertile of plasma adiponectin level or VAT/SAT. The efficacy of VAT/SAT and adiponectin to predict cerebral atherosclerosis was assessed using the receiver-operating characteristic (ROC) curve. To distinguish their prediction powers, researchers employed the MedCalc statistical software. The value *p* < 0.05 was regarded as statistically significant, and SPSS v25 was used for statistical analysis (IBM Corporation, New York, United States).

## Results

Due to a lack of key clinical data, a history of severe abdominal organ disease or previous abdominal surgery, 40 patients were removed from the data analysis. Finally, 186 patients (59.1% men; mean age: 67.19 ± 8.68 years) were enrolled in the study, including the non-ICAS group (*n* = 91; 48.9%) and the ICAS group (*n* = 95; 51.1%) (Table 1). When compared to the non-ICAS group, patients in the ICAS group had a larger VAT/SAT ratio (1.15 ± 0.30 vs. 0.89 ± 0.25, *p* < 0.001), a greater prevalence of NAFLD (37.9% vs. 19.8%, *p* = 0.007), a higher prevalence of diabetes (55.8% vs. 30.8%, *p* = 0.001), higher elevated fibrinogen levels (2.96 ± 1.03 vs. 2.55 ± 0.77, *p* = 0.002), and lower adiponectin levels (2.43 ± 0.99 vs. 3.36 ± 1.27, *p* < 0.001). However, there was no statistical significance in coronary heart disease, carotid intima-media thickness (IMT), carotid plaque, hypertension, triglyceride level, or LDL-C level between the two groups (*p*>0.05 for all). The lipid ratio is considered a good parameter for identifying ICAS (24). Referring to their research, we also included LDL-C/HDL-C, TC/HDL-C, TG/HDL-C, non-HDL-C/HDL-C in the analysis, and there was no statistical difference between the two groups (Table 1). Univariate analysis revealed that the ICAS group had a substantially larger abdominal perimeter, VAT area, and VAT/SAT than the non-ICAS group [90.77 ± 10.37 vs. 87.69 ± 10.61, *p* = 0.046; 184.53 ± 57.58 vs. 154.27 ± 52.30, *p* < 0.001; 1.15 ± 0.30 vs. 0.89 ± 0.25, *p* < 0.001] (Table 1). In multivariate logistic regression analysis, after controlling for potential confounders, we discovered that the VAT/SAT ratio (OR, 26.08; 95% CI, 5.92–114.83; *p* < 0.001), NAFLD (OR, 2.95; 95% CI, 1.33–6.50; *p* = 0.008), fibrinogen levels (OR, 1.80; 95% CI, 1.12–2.88; *p* = 0.014), diabetes mellitus (OR, 2.51; 95% CI, 1.22–5.18; *p* = 0.013), and comparatively low adiponectin levels (OR, 0.61; 95% CI, 0.44–0.84; *p* = 0.002) were independent predictors of increased ICAS risk (Table 2).

**Table 1:** Clinical characteristics and abdominal adipose-relevant parameters of patients.

**Variable**	**Total (*n* = 186)**	**Non-ICAS (*n* = 091)**	**ICAS (*n* = 95)**	***P*** **value**
Age (year)	67.19 ± 8.68	66.73 ± 8.31	67.64 ± 9.04	0.472
Male, *n* (%)	110 (59.1)	45 (49.4)	65 (68.4)	0.009
TAT area (cm^2^)	341.66 ± 96.57	333.16 ± 95.26	349.80 ± 97.62	0.241
VAT area (cm^2^)	169.72 ± 56.97	154.27 ± 52.30	184.53 ± 57.58	<0.001
SAT area (cm^2^)	171.94 ± 53.91	178.89 ± 56.74	165.27 ± 50.45	0.086
VAT/SAT ratio	1.02 ± 0.31	0.89 ± 0.25	1.15 ± 0.30	<0.001
BMI (kg/ cm^2^)	24.80 ± 2.01	24.65 ± 2.01	24.95 ± 2.02	0.309
Abdominal circumference (cm)	89.26 ± 10.57	87.69 ± 10.61	90.77 ± 10.37	0.046
NAFLD, *n* (%)	54 (29)	18 (19.8)	36 (37.9)	0.007
Smoking, *n* (%)	64 (34.4)	25 (27.5)	39 (41.1)	0.051
Drinking, *n* (%)	38 (20.4)	17 (18.7)	21 (22.1)	0.563
Diabetes mellitus, *n* (%)	81 (43.5)	28 (30.8)	53 (55.8)	0.001
Coronary heart disease, *n* (%)	24 (12.9)	11 (12.1)	13 (13.7)	0.745
Carotid intima-media thickness (mm)	0.79 ± 0.03	0.79 ± 0.03	0.80 ± 0.02	0.113
Carotid plaque, *n* (%)	98 (52.6)	44 (48.4)	54 (56.8)	0.246
Hypertension, *n* (%)	148 (79.6)	68 (74.7)	80 (84.2)	0.109
Triglyceride (mmol/L)	1.60 ± 1.04	1.58 ± 1.01	1.62 ± 1.08	0.822
Total cholesterol (mmol/L)	3.51 ± 1.47	3.64 ± 1.48	3.39 ± 1.46	0.250
HDL-C (mmol/L)	1.47 ± 0.95	1.54 ± 0.91	1.40 ± 1.00	0.284
LDL-C (mmol/L)	2.59 ± 1.01	2.67 ± 0.94	2.51 ± 1.06	0.268
Non-HDL-C (mmol/L)	2.04 ± 2.07	2.09 ± 2.05	2.00 ± 2.09	0.744
Creatinine (mmol/L)	76.84 ± 31.42	70.82 ± 35.51	82.60 ± 25.82	0.011
Uric acid (mmol/L)	337.20 ± 91.53	324.71 ± 84.90	349.16 ± 96.40	0.068
Fibrinogen (mmol/L)	2.76 ± 0.93	2.55 ± 0.77	2.96 ± 1.03	0.002
Glycated hemoglobin (%)	6.48 ± 1.21	6.30 ± 1.10	6.66 ± 1.28	0.042
Fasting glucose (mmol/L)	6.02 ± 1.90	5.85 ± 1.83	6.18 ± 1.96	0.234
Adiponectin (ug/ml)	2.89 ± 1.23	3.36 ± 1.27	2.43 ± 0.99	<0.001
LDL-C/HDL-C	2.11 ± 1.06	2.06 ± 1.01	2.16 ± 1.11	0.549
TC/HDL-C	3.09 ± 1.64	3.03 ± 1.57	3.15 ± 1.72	0.605
TG/HDL-C	1.42 ± 1.52	1.35 ± 1.35	1.48 ± 1.68	0.553
Non-HDL-C/HDL-C	2.09 ± 1.64	2.03 ± 1.57	2.15 ± 1.72	0.606

*Data are presented as mean ± standard deviation or median (interquartile range) and numbers (percentage)*.

*TAT, Total adipose tissue; VAT, Visceral adipose tissue; SAT, Subcutaneous adipose tissue; BMI, Body mass index; NAFLD, Nonalcoholic fatty liver disease; HDL-C, High-density lipoprotein cholesterol; LDL-C, Low-density lipoprotein cholesterol; ICAS, Intracranial atherosclerotic stenosis*.

**Table 2:** Univariate and multivariate analysis of the risk factors for ICAS.

**Variables**	**Univariate**		**Multivariate**	
	**OR (95% CI)**	***P***	**OR (95% CI)**	***P***
Male, *n* (%)	2.22 (1.22–4.02)	0.009	–	–
VAT area (cm^2^)	1.01 (1.00–1.02)	<0.001	–	–
VAT/SAT ratio	37.45 (9.97–140.61)	<0.001	26.08 (5.92–114.83)	<0.001
Abdominal circumference (cm)	1.03 (1.00–1.06)	0.048	–	–
NAFLD, *n* (%)	2.48 (1.28–4.80)	0.007	2.95 (1.33–6.50)	0.008
Diabetes mellitus, *n* (%)	2.84 (1.56–5.18)	0.001	2.51 (1.22–5.18)	0.013
Creatinine (mmol/L)	1.02 (1.00–1.03)	0.016	–	–
Fibrinogen (mmol/L)	1.76 (1.20–2.57)	0.003	1.80 (1.12–2.88)	0.014
Glycated hemoglobin (%)	1.29 (1.00–1.66)	0.046	–	–
Adiponectin (ug/ml)	0.49 (0.37–0.65)	<0.001	0.61 (0.44–0.84)	0.002

*P < 0.05 as assessed by the univariate logistic regression, variable was subsequently included in the multivariate analysis*.

*VAT, Visceral adipose tissue; SAT, Subcutaneous adipose tissue; NAFLD, Nonalcoholic fatty liver disease*.

**Figure 1:**
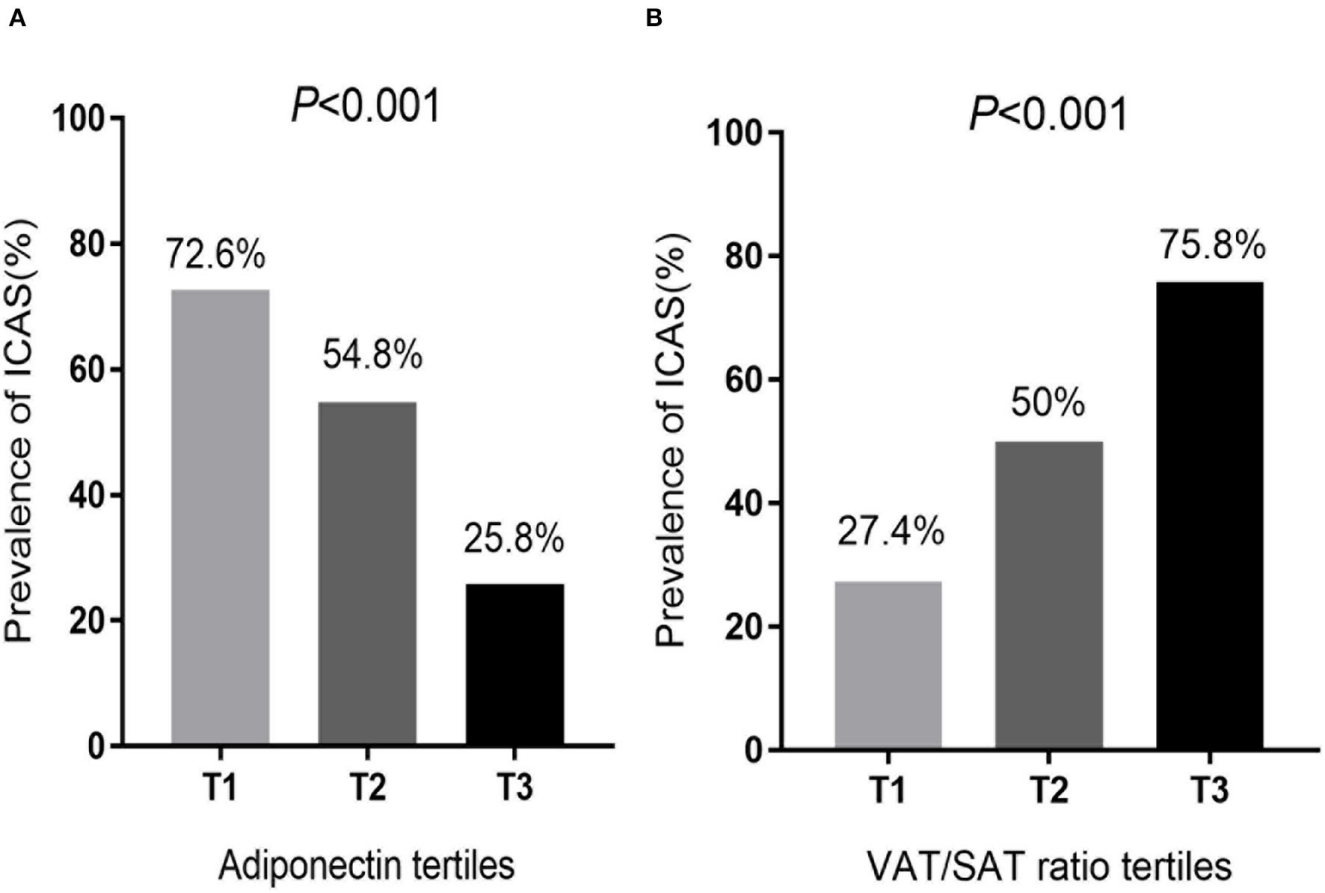
Comparison of **(A)** adiponectin tertiles and **(B)** VAT/SAT ratio tertiles determined according to the prevalence of ICAS. **(A)** Participants were divided into 3 groups, T1: low-adiponectin (0.83–2.09 ug/ml), T2: middle-adiponectin (2.10–3.41 ug/ml), and T3: high-adiponectin (3.42–6.38 ug/ml). **(B)** Participants were divided into 3 groups, T1: low-VAT/SAT (0.30–0.84), T2: middle-VAT/SAT (0.85–1.13), and T3: high-VAT/SAT (1.14–2.06). VAT, Visceral adipose tissue; SAT, Subcutaneous adipose tissue; ICAS, Intracranial atherosclerotic stenosis.

We used tertiles to categorize all VAT/SAT ratios and adiponectin levels to further investigate the relationship between ICAS, VAT/SAT, and adiponectin (Figure 1). The prevalence of ICAS increased (T1: 27.4%; T2: 50.0%; T3: 75.8%) as VAT/SAT ratio tertile increased (*p* < 0.001). The prevalence of ICAS decreased (T1: 72.6%; T2: 54.8%; T3: 25.8%) as adiponectin tertile increased (*p* < 0.001).

**Figure 2:**
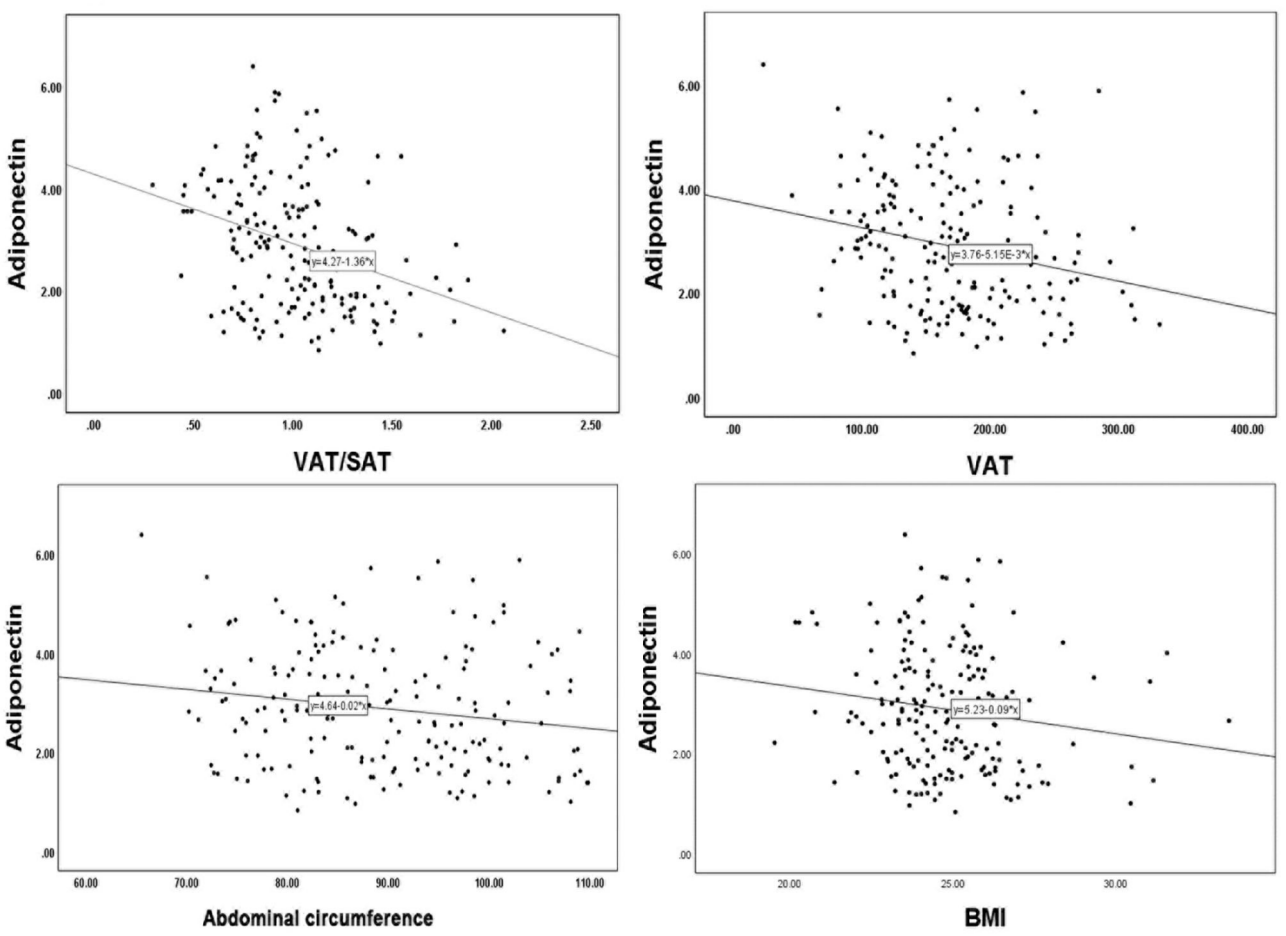
Linear correlation analysis of adiponectin with VAT/SAT, VAT, abdominal circumference, BMI. VAT, visceral adipose tissue; SAT, subcutaneous adipose tissue; BMI, body mass index.

To investigate the relationship between adiponectin and obesity, we performed a Spearman correlation analysis of adiponectin and abdominal fat-related parameters (Figure 2; Table 3). VAT/SAT ratio (R = −0.337, *p* < 0.001) was negatively correlated with adiponectin compared with VAT (R = −0.239, *p* = 0.001), abdominal circumference (R = −0.170, *p* = 0.021), and BMI (R = −0.155, *p* = 0.034).

**Table 3:** Spearman correlation analyses between adiponectin and VAT/SAT, VAT, abdominal circumference, and BMI.

**Variables**	**R**	***P*** **value**
VAT/SAT ratio	−0.337	<0.001
VAT	−0.239	0.001
Abdominal circumference	−0.170	0.021
BMI	−0.155	0.034

*VAT, Visceral adipose tissue; SAT, Subcutaneous adipose tissue; BMI, Body mass index*.

**Figure 3:**
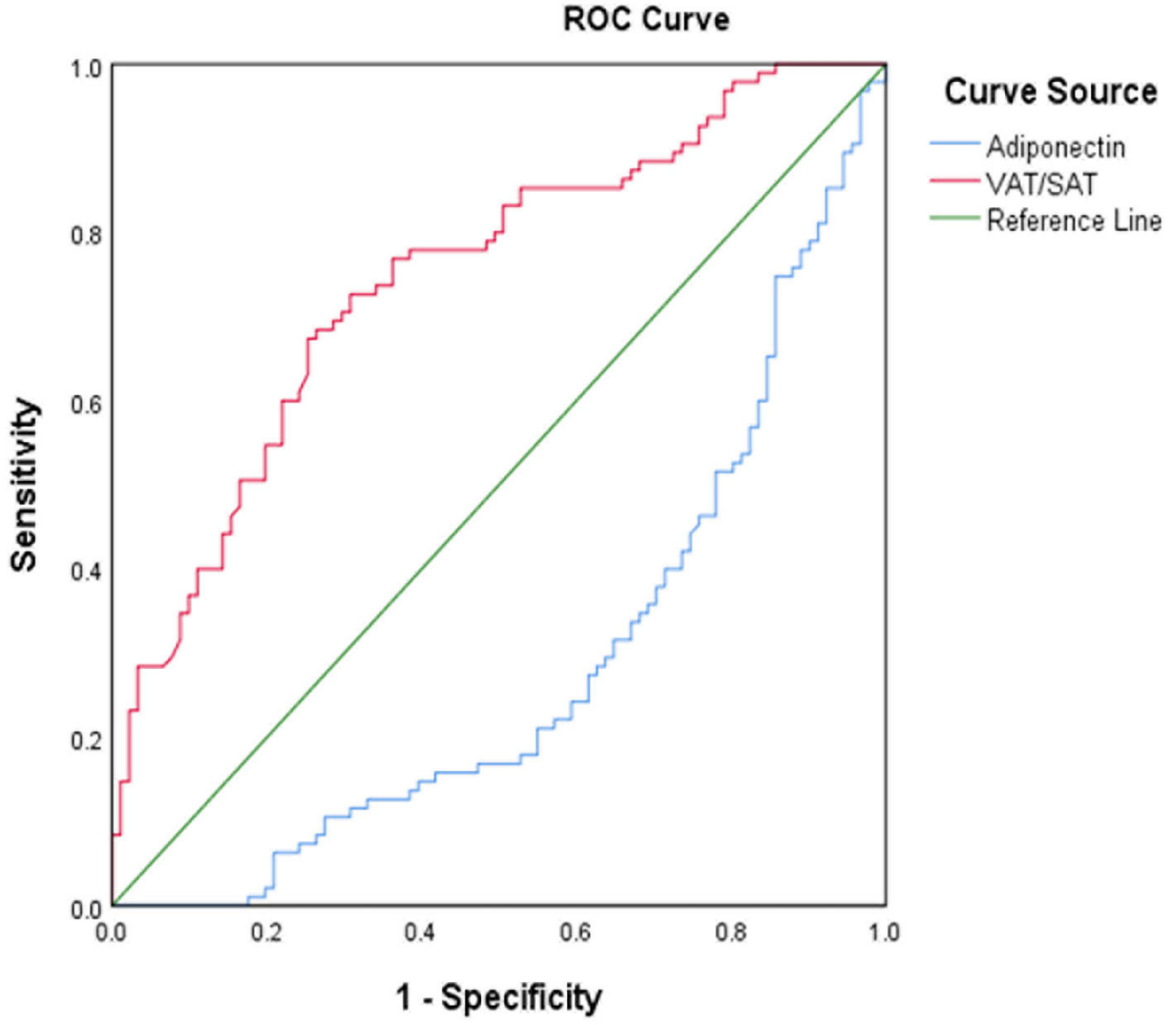
ROC curve for VAT/SAT and adiponectin for distinguish between ICAS and non-ICAS. ROC, receiver operating characteristic curve; VAT, visceral adipose tissue; SAT, subcutaneous adipose tissue; ICAS, intracranial atherosclerotic stenosis.

The ROC curve analysis illustrated the distinctive ability of independent risk factors to predict ICAS (Figure 3; Table 4). VAT/SAT ratio had sensible accuracy for the prediction of ICAS (AUC: 0.747; *p* < 0.001; sensitivity: 67.4%; specificity: 74.7%). The optimal cut-off value of VAT/SAT ratio was 1.04. The optimal adiponectin cutoff to predict ICAS in this study was 3.03 ug/ml (AUC: 0.716; *p* < 0.001; sensitivity: 75.8%; specificity: 61.5%).

**Table 4:** ROC curve for predicting ICAS and cutoff points for maximum sum of sensitivity and specificity.

**Variables**	**ROC (95%CI)**	**Cutoff point**	**Sensitivity (%)**	**Specificity (%)**
VAT/SAT	0.75 (0.64–0.79)	1.04	67.4	74.7
Adiponectin	0.72 (0.68–0.82)	3.03	75.8	61.5

*VAT, Visceral adipose tissue; SAT, Subcutaneous adipose tissue*.

## Discussion

In this study, we explored the relationships between abdominal visceral fat content and clinical characteristics in non-ICAS and ICAS patients. We found that male gender, VAT area, VAT/SAT ratio, abdominal circumference, diabetes mellitus, creatinine level, fibrinogen level, glycated hemoglobin level, NAFLD, and adiponectin level were predictors of ICAS. VAT/SAT, NAFLD, diabetes mellitus, fibrinogen level, and lower adiponectin level were found to be independent risk factors for ICAS in a multivariable logistic regression analysis. Obesity is considered a risk factor for atherosclerosis (25). VAT contributed not only to general but also to the advancement of subclinical atherosclerosis, especially in ladies. Both previous research and our findings indicate that the particular part of VAT participated in the early advancement of atherosclerosis (26). In a general population sample from Norway, adiposity has consistently been associated with carotid atherosclerotic plaque burden (27). A small number of studies have investigated the association of BMI with ICAS, although they failed to demonstrate a significant association. However, as a measure of adiposity, BMI has limitations. Abdominal circumference is a sign of abdominal obesity, which is more closely related to subclinical atherosclerosis than BMI (28). Abdominal adiposity is associated with insulin resistance and atherosclerosis (29). It is speculated that the increased risk of abdominal obesity may be caused by the accumulation of large amounts of visceral fat tissue. However, abdominal parameters cannot be used to distinguish VAT from SAT, as the former has different biological characteristics and functions (30). Hence, we measured the abdominal fat including TAT, VAT, and SAT using abdominal CT. Previous research has suggested that the absolute value of VAT and SAT, as well as their ratio, may play a significant role in the progression of abdominal atherosclerosis (26). In our study, there was no statistical difference between the two groups in terms of the BMI, TAT area, and SAT.

Notably, VAT area, abdominal circumference, and VAT/SAT ratio are higher in the ICAS group than in the non-ICAS group. After adjusting the traditional cardiovascular risk factors, the VAT/SAT ratio remains an independent predictor. Previous studies have shown that VAT is metabolically active and causes chronic systemic inflammation through the secretion of adipokines, which is an important cause of insulin resistance caused by obesity (31, 32). Several studies have indicated that SAT has a protective effect against insulin sensitivity. When compared to the absolute value of VAT or SAT, the VAT/SAT ratio may be a better predictor of abdominal fat distribution.

Clinical research published over the last few decades has shown higher fibrinogen levels in people with atherosclerotic disease (33). Serum fibrinogen levels over a certain threshold may raise the risk of vascular disease by increasing viscosity, stimulating fibrin formation, or increasing platelet interaction. More inflammatory cytokines are produced as a result of a dysfunctional interaction between fat cells and tissue macrophages, which is linked to obesity (33, 34). Fibrinogen is closely linked with subclinical atherosclerosis in obese individual (35). Increased fibrinogen levels throughout time are closely linked to the occurrence of early carotid lesions, notably plaques. Recent studies have shown that in the Han population, higher fibrinogen levels are also independently related to the risk and severity of coronary atherosclerosis (36). We discovered that a high fibrinogen level was an independent predictor of ICAS in this study. Fibrinogen is a powerful predictor of ischemic stroke, according to some studies, although it does not indicate the type or clinical outcomes of stroke (37). We suggest that high fibrinogen levels may be a predictor of a large atherosclerotic stroke.

Adiponectin is an adipocyte secretory protein whose levels in the blood are reduced in obese and diabetic individuals. It may be beneficial to atherosclerosis, inflammation, insulin resistance pathways, and lipid disorders, making it an appealing biomarker and therapeutic target for cardiometabolic diseases such as coronary heart disease, non–cardiac vascular diseases, MetS, NAFLD, and T2DM (15). Okauchi et al. pointed out that the serum adiponectin concentration is related to BMI, WC, and VAT of middle-aged people (38). The concentration of adiponectin in circulation is negatively correlated with body fat, especially visceral fat (39). In our study, we found that VAT/SAT ratio was more negatively correlated with adiponectin than VAT, BMI, and abdominal circumference (Figure 2; Table 3). Many studies showed that visceral fat accumulation and decreased plasma adiponectin concentration were associated with insulin sensitivity, insulin resistance, and vascular inflammation (40–42). Patients with large artery atherosclerosis (LAA) strokes reported lower levels of serum adiponectin than those who experienced non-LAA strokes (43). Bang OY et al. found that adiponectin levels were associated with intracranial atherosclerosis. Moreover, adiponectin levels varied by stroke subtype, with the ICAS group having the lowest (44). Their results are similar to our current findings that the lower plasma adiponectin levels were related to the increased risk of intracranial atherosclerotic stenosis (ICAS). Here, we find that besides adiponectin, the VAT/SAT ratio appears to have a strong predictor for ICAS. Although a majority of clinical studies analyzed and compared total adiponectin levels and found the potential role of adiponectin in cardio-cerebrovascular diseases including cerebral infarction disease, it is also important to further explore the role of each isoform of adiponectin.

Adiponectin as a secreted multimeric protein has three isoforms: low molecular weight (LMW), medium molecular weight (MMW), and high molecular weight (HWM) (45). HMW adiponectin is the most active oligomeric form of adiponectin. There is evidence that circulating levels of total and HWM adiponectin are associated with coronary heart disease, ischemic stroke, and peripheral arterial disease (46). Besides, the research reported a positive association between LMW adiponectin and all-cause mortality in the elderly, which was thought contingent on unbalanced circulating levels of adiponectin isoforms (47). Noriko Tagawa et al. found that decreased total adiponectin, HMW adiponectin, and LMW adiponectin may be associated with atherosclerotic infarction, and that serum LMW adiponectin level was a credible biomarker for cerebrovascular disorders (48).

There are several limitations that need to be emphasized. Firstly, longitudinal observations are required to evaluate the longitudinal effects of visceral fat in the progression of ICAS. Secondly, because of the small sample size, there was no further precise classification of the atherosclerosis stenosis and ≥50% was used to determine significant stenosis in this study. Thirdly, previous studies have shown that the apo B/apo A-I ratio is considered to be an excellent surrogate for the prediction of vascular disease risk, such as ICAS (24, 49). Initially we wanted to include this parameter in the study, but for some economic reasons, our center does not routinely screen the two indicators of apo B and apo A-I. Such lipid parameters need to be further explored in a clinical or community study with a larger sample. Finally, the relationship between different isoforms of adiponectin and ICAS has not been studied here partly due to the difficulty in measuring each adiponectin isoform, and we will explore it in the future.

## Conclusion

In conclusion, the higher VAT/SAT ratio and lower plasma adiponectin levels were closely related to the increased risk of ICAS. The traditional cardiovascular risk factor, diabetes mellitus, is still considered an independent predictor of ICAS. Adiponectin may have a protective effect against atherosclerosis. The VAT/SAT ratio appears to have a strong predictive ability for ICAS.

## Data Availability Statement

The raw data supporting the conclusions of this article will be made available by the authors, without undue reservation.

## Ethics Statement

The studies involving human participants were reviewed and approved by Ethics Committee of Shanghai Tenth People's Hospital. The patients/participants provided their written informed consent to participate in this study.

## Author Contributions

Y-XZ, J-CX, and Q-KB conceived and designed the study. HQ and J-YH collected the clinical data. T-YY performed statistical analyses. F-HW and L-YM analyzed the data and drafted the paper and YT helped. All authors read and approved the final manuscript. All authors contributed to the article and approved the submitted version.

## Funding

The present study was supported by the Science and Technology Commission of Shanghai Municipality (Grant Number: 20ZR1443500), the Featured Clinical Discipline Project of Shanghai Pudong (PWYst2018-01), Key Discipline Group Construction Project of Shanghai Pudong (PWZxq2017-02), the Clinical Research Project of Shanghai Tenth People's Hospital (YNCR2C030), and Clinical Research Project of Shanghai Municipal Health Commission (201940452).

## Conflict of Interest

The authors declare that the research was conducted in the absence of any commercial or financial relationships that could be construed as a potential conflict of interest.

## Publisher's Note

All claims expressed in this article are solely those of the authors and do not necessarily represent those of their affiliated organizations, or those of the publisher, the editors and the reviewers. Any product that may be evaluated in this article, or claim that may be made by its manufacturer, is not guaranteed or endorsed by the publisher.
